# Miscellaneous

**Published:** 1889-07

**Authors:** 


### MISCELLANEOUS.

Diphtheria from Animals.—Further instances come—also from Italy—additional to the striking reports of infection from fowls and cats, some time ago reproduced in the *Sanitary Era*. Dr. Cazenave de la Roche publishes an article on the subject in the "Revue generale de clinique et de therapeutique," for January 2, 1889. Called to attend two young women suffering from diphtheria of abnormal virulence, his suspicions were directed toward a pigeon-house and a hen-house near the cistern which provided the drinking water for the family. A few days before the disease manifested itself, a violent rain-storm had washed the roofs of these out-buildings, and carried the fæcal deposits into the cistern, and this was the only conceivable source of infection in the case. Twenty years ago, while in Venice, the author had noticed the prevalence of diphtheria in the hospital of San Juan y Paolo. He considers that the disease was due to the contamination of the cisterns by the numerous pigeons of that city.

All who keep birds or fowls should be mindful to guard against their excrement as a dangerous contaminator of water into which it may be washed by rain on the roofs, trees, or roosts which they frequent. Never had diphtheria from that source? Then perhaps you had better wait until you lose a few children, before paying any attention to the matter. That is the common-sense way—as generally understood.—*The Sanitary Era.*

The negro's black cuticle has long baffled physiologists. It is now asserted that its function is to to convert the sun's light into heat, most of which it holds. The true sensitive skin underneath, as well as the negro's whole interior is thus protected. Encased, as it were, in this sun-proof armor, negroes stand heat far better than white men, and seldom have sunstroke.

To Wash Chamois-Leather Gloves.—Put the gloves on the hands, and wash the same as if one was washing the hands. When clean, take them off under the water as that keeps them in shape; wring dry in a towel, open with a glove-stretcher, and hang them up to dry. If the directions are followed, they will look as nice as new.

Music in Medicine.—From the time when medical knowledge was first embodied in rules of practice, and probably from a much earlier period, music has held a recognized place in the treatment of disease. In no classes of diseases, however, are we likely to derive so much benefit from the use of so pleasant a remedy as in those affecting the mind itself. In melanancholia and allied estates of depression its value is generally admitted in our own day. Ancient practitioners were also cognizant of its usefulness in this respect. We must all have felt how suitable is its infinite variety and facility of expression to the changing moods of the sane, and it is, therefore, the less difficult to understand how straying minds are pleased and settled by its charms.—*London Lancet.*

The Human Breath.—Professor Brown-Sequard has recently been making experiments to determine whether the human breath was capable of producing any poisonous effects. From the condensed watery vapor of the expired air he obtained a poisonous liquid, which, when injected under the skin of rabbits, produced almost immediate death. He ascertained that this poison was an alkaloid, and not a microbe. The rabbits thus injected died without convulsions, the heart and large blood vessels being engorged with blood. Brown-Sequard considers it fully proved that the expired air, both of man and animals, contains a volatile poisonous principle which is much more deterious than carbonic acid.

A GERMAN patent for a fire-and-waterproof paper was lately granted to M. zinc Ladewigg. The process is as follows: Twenty-five parts of asbestos are mixed with from twenty-five to thirty parts of alum, and moistened with a solution of chloride, and well washed in running water. The pulp is then treated with a solution of one part of resin soap and eight or ten parts of pure alum, after which it is made into paper exactly as rag pulp is. The result is a paper which resists the action of both fire and water.

RED TAPE IN RUSSIA.—From an illustrated article by George Kennan entitled "Exile by Administrative Process," in the Century Magazine, we quote as follows: "How easy it is in Russia to get a high official's signature to any sort of a document may be illustrated by an anecdote that I have every reason to believe is absolutely true. A 'stola-nachalnik,' or head of a bureau, in the provincial administration of Tobolsk, while boasting one day about his power to shape and direct governmental action, made a wager with another chinovnik that he could get the governor of the province—the late Governor Lissogorski—to sign a manuscript copy of the Lord's Prayer. He wrote the prayer out in the form of an official document on a sheet of stamped paper, numbered it, attached the proper seal to it, and handed it to the governor with a pile of other papers which required signature. He won his wager. The governor duly signed the Lord's Prayer, and it was probably as harmless an official document as ever came out of his office."

